# Draft Genome Sequence of *Aeromonas caviae* 8LM, Isolated from Stool Culture of a Child with Diarrhea

**DOI:** 10.1128/genomeA.00524-15

**Published:** 2015-05-21

**Authors:** Bárbara Moriel, Leonardo M. Cruz, Cibelle B. Dallagassa, Helisson Faoro, Emanuel M. de Souza, Fábio O. Pedrosa, Fabiane G. M. Rego, Geraldo Picheth, Cyntia M. T. Fadel-Picheth

**Affiliations:** a Departamento de Análises Clínicas, Universidade Federal do Paraná, Curitiba, PR, Brazil; b Departamento de Bioquímica e Biologia Molecular, Universidade Federal do Paraná, Curitiba, PR, Brazil

## Abstract

*Aeromonas* spp. are Gram-negative rods ubiquitous in aquatic environments; however, some species are able to cause a variety of infections in humans. Here, we report the draft genome sequence of *Aeromonas caviae* 8LM isolated from stool culture from a child with diarrhea in southern Brazil.

## GENOME ANNOUNCEMENT

*Aeromonas* spp. are Gram-negative rods associated with a variety of infections in humans (1), gastroenteritis being the most common (2). In our geographical region *Aeromonas*-associated diarrhea is common, *Aeromonas caviae* being the predominant species (3, 4). Here, we report the draft genome sequence of *A. caviae* 8LM, recovered from the stool culture of a 9-month-old child with diarrhea attended by Sistema Único de Saúde at Curitiba in southern Brazil.

Sequencing was performed using paired-end sequencing with an Illumina Genome Analyzer IIx. Analysis and data processing were performed with CLC Genomic Workbench v6.5.1 for assembly of contigs. Automatic genome annotation was performed by Rapid Annotations using Subsystems Technology (RAST) (5). The genome assembly revealed a length of 4,477,475 bp at 385× coverage, an *N*_50_ of 369,568 bp, allocated into 34 contigs, a G+C content of 61.8%, 117 RNAs, and 4,076 CDSs. The G+C content and size are similar to those described for *A. caviae* Ae398 (61.4% and 4.43 Mb, respectively) (6).

Regarding antimicrobial resistance, genes coding for β-lactamase class D, aminoglycoside N3′-acetyltransferase, multidrug-resistant efflux pumps, chloramphenicol acetyltransferase, and FosA for fosfomycin resistance were found. Some genes coding for virulence traits, such as hemolysins, phospholipase/lecithinase, RtxA toxin, polar flagellum, type IV pilus, and a type 6 secretion system, were found. Additionally, genes coding for choloylglycine hydrolase and DamX associated with bile hydrolysis or resistance, respectively, were found and may facilitate colonization of the intestine by the 8LM strain. The genome sequence of *A. caviae* 8LM will contribute to the understanding of the mechanisms used by the bacteria to survive, colonize the intestine, and cause diarrhea.

### Nucleotide sequence accession numbers.

This whole-genome sequence has been deposited at DDBJ/EMBL/GenBank under the accession no. LAFH00000000. The version described in this paper is version LAFH01000000.
